# Calculating the Effect of Ribs on the Focus Quality of a Therapeutic Spherical Random Phased Array

**DOI:** 10.3390/s21041211

**Published:** 2021-02-09

**Authors:** Muhammad Zubair, Robert Dickinson

**Affiliations:** 1 Department of Radiation Oncology, University of California, San Francisco, CA 90007, USA; 2 Department of Bioengineering, Imperial College London, London SW7 2AZ, UK; robert.dickinson@ic.ac.uk

**Keywords:** random phased array, transcostal, therapy, HIFU, rib cage, ultrasound intensity, focus quality

## Abstract

The overlaying rib cage is a major hindrance in treating liver tumors with high intensity focused ultrasound (HIFU). The problems caused are overheating of the ribs due to its high ultrasonic absorption capability and degradation of the ultrasound intensity distribution in the target plane. In this work, a correction method based on binarized apodization and geometric ray tracing approach was employed to avoid heating the ribs. A detailed calculation of the intensity distribution in the focus plane was undertaken to quantify and avoid the effect on HIFU beam generated by a 1-MHz 256-element random phased array after the ultrasonic beam passes through the rib cage. Focusing through the ribs was simulated for 18 different idealized ribs-array configurations and 10 anatomically correct ribs-array configurations, to show the effect of width of the ribs, intercostal spacing and the relative position of ribs and array on the quality of focus, and to identify the positions that are more effective for HIFU applications in the presence of ribs. Acoustic simulations showed that for a single focus without beam steering and for the same total acoustic power, the peak intensity at the target varies from a minimum of 211 W/cm^2^ to a maximum of 293 W/cm^2^ for a nominal acoustic input power of 15 W, whereas the side lobe level varies from 0.07 I_peak_ to 0.28 I_peak_ and the separation between the main lobe and side lobes varies from 2.5 mm to 6.3 mm, depending on the relative positioning of the array and ribs and the beam alignment. An increase in the side lobe level was observed by increasing the distance between the array and the ribs. The parameters of focus splitting and the deterioration of focus quality caused by the ultrasonic propagation through the ribs were quantified in various possible different clinical scenarios. In addition to idealized rib topology, anatomical realistic ribs were used to determine the focus quality of the HIFU beam when the beam is steered both in axial and transverse directions and when the transducer is positioned at different depths from the rib cage.

## 1. Introduction

Hepatocellular Carcinoma (HCC) is the most common form of liver cancer and one of the leading causes of cancer-related deaths [1]. Treatment options include liver transplantation and surgery; however, only about 25% of patients are suitable for surgical resection, depending on the size and location of the tumor. Moreover, resection is an invasive procedure and has the usual risks associated with surgery.

High intensity focused ultrasound (HIFU) is a non-invasive medical procedure that uses high-amplitude waves to heat and ablate the tumor. The tumor is destroyed by accurately targeting acoustic energy deep into the tissue without damaging the intervening tissue. The feasibility of HIFU for treatment of a range of tumors including liver cancer has been demonstrated in several studies [2,3,4]. However, there are several challenges that hinder its widespread clinical application. One of the major limitations is the presence of intervening structures such as rib cage that obstruct the acoustic beam path [5,6]. Three problems are associated with the presence of rib cage in the HIFU beam path. First, absorption of acoustic energy by the ribs causing local heating and skin burns [5]. Secondly, reduction in the intensity at the focal spot due to the shadowing of elements of the phased array by the ribs [7], and, finally, focus deterioration due to the periodic structure of the ribs [8].

Several measures have been proposed to minimize heating of the ribs while preserving high intensity at the focus in the presence of rib cage. Surgical removal of the ribs that intersect with the HIFU beam path was proposed by Wu et al. [6]. Botros et al. [9] adjusted the amplitudes and phases applied to a spherical phased array in theoretical studies to reduce the exposure of the ribs. Civale et al. [10] used a linearly segmented transducer for intercostal sonications to lower the temperature on the ribs. A numerical study was performed by Liu et al. [5] in which the array elements being obstructed by the ribs were deactivated to minimize heat deposition over the ribs. Their approach is based on geometric ray-tracing using computed tomography (CT) images of the rib cage in which elements obstructed by the ribs are deactivated leading to a reduction in undesired heating. Quesson et al. [11] used this approach in in-vivo experiments by using anatomical data derived from magnetic resonance (MR) images. The approach was further validated and found effective in other studies [7,12,13]. Another beam shaping method called phase conjugation method, based on a time-reversal technique [12], was introduced by Aubry et al. [13]. In this method, the signal received by each transducer element from a point source positioned in the focus is re-emitted in a time-reversed manner. The focus is thus created at the target location while avoiding energy exposure on the ribs. An inverse problem using the boundary element method (BEM) was developed to optimize the apodization of the array elements by taking into account physical effects such as scattering and diffraction [14]. The drawbacks of these techniques are that they are either not completely non-invasive or they rely on an anatomical model based on CT or MR images for apodization of the array elements and are computationally expensive. The CT images that are used to build the anatomical 3D model are acquired with the patient not in the final treatment position and thus are not clinically valid due to local anatomy deformations. Although the MR images represent accurate spatial representation of the target site and surrounding anatomy when taken with the patient in the treatment position, a long segmentation process is required to transform the anatomical images into a 3D model in order to determine the transducer apodizations.

To account for these limitations, other methods that do not rely on an external imaging modality were suggested. Cochard et al. [15,16] used a method based on decomposition of a time-reversal operator (DORT) by analyzing backscattered echoes from ex-vivo ribs immersed in water and deactivated the blocked elements in order to minimize heat on the ribs. This technique, however, requires the presence of strong well-resolved point-like scatterers unlike ribs which have a complex anatomical shape. Marquet et al. [17] used a simple pulse-echo detection technique where the strength of the amplitude of back-scattered signals was used to determine the location of the ribs. This technique is fast but has the limitation of not distinguishing between echoes from ribs and other tissues due to the inherent limitation of the therapeutic transducer. A rib detection method based on cavitation-enhanced back projection was introduced by Ramaekers et al. [18] in which attenuation between each transducer element and the focus is determined based on the strength of the signal received from the bubble cloud produced by emitting a short pulse through all transducer elements simultaneously. Unlike the techniques used by Aubry et al. [13] and Cochard et al. [16], this method did not require the presence of a point-like scatterer at the focus; however, the induction of stable cavitation deep in the tissue requires delivery of large acoustic power which not only increase the complexity of the system but may also pose a risk of undesired tissue damage due to non-linear energy deposition.

The effect of rib cage on focus deterioration has also been subject of some studies. Bobkova et al. [7] determined that the rib cage acts as diffractor and quantified the parameters of focus splitting. Khokhlova et al. [8] theoretically investigated focus splitting associated with the propagation of ultrasound energy through the rib cage. Almekkaway et al. [19] used a semidefinite relaxation technique for optimal refocusing through the ribs and to control the side lobe level in the focal plane. These studies were however limited to idealized 2D rib phantoms and the effects of a realistic rib cage on the quality of focus were not considered.

The random distribution of the elements on the spherical array has made it possible to steer the beam electronically without creating grating lobes, however, the effect of steering the focus on focus quality and the creation of side lobes in the presence of realistic ribs has not hitherto been studied in detail. A thorough understanding of the effect of rib cage on ultrasonic pressure field of the HIFU array is required as part of the treatment planning strategy. The goal of any treatment planning will be to achieve high peak intensity levels in the focal plane and low intensity levels in the plane of the ribs. We previously demonstrated that the therapeutic random phased array can be used to produce 3D images of intervening structures such as ribs [20,21]. The images produced can then be used for refocusing the HIFU energy in the presence of rib cage by selectively deactivating elements which are shadowed by the ribs, thus minimizing depositing acoustic energy on the ribs. The effect of the ribs on the quality of the focus was however not determined and is the subject of this study.

The aim of this study is to find strategies to improve the focusing of ultrasonic energy in transcostal applications and to analytically investigate the effect of the rib cage on the beam distortion of the HIFU system, calculate intensity distribution, and perform parametric evaluation by analyzing focus quality for various parameters and in different scenarios. A binarized apodization technique based on a geometric ray tracing approach [5,11] was used in a simulation in the presence of an idealized as well as a patient-specific rib cage model to study and thereafter minimize the effect of HIFU on the ribs. Intensity distribution was calculated in the plane of the ribs and in the focal plane. Various scenarios that can arise during the HIFU procedure were simulated to analyze focus splitting for different parameters of array-rib configurations, rib cage dimensions, and position of the array with respect to the rib cage. The results presented here provide both qualitative and quantitative information about the HIFU beam distortion in the presence of a rib cage and can be useful for treatment planning and clinical application of HIFU treatment of liver cancer.

## 2. Materials and Methods

### 2.1. The Random Phased Array Transducer

**Figure 1:**
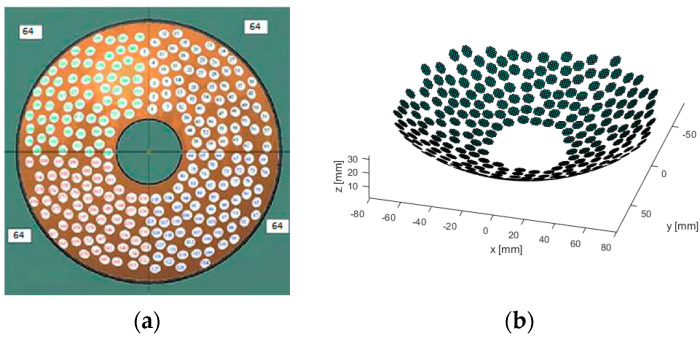
The random distribution of elements in four quadrants with 64 elements in each quadrant. The diameter of the array is 180 mm and the central hole is 38 mm in diameter. (**a**) Front face, (**b**) 3D view.

The array transducer (Imasonics, Vorayr surl’Ognon, France) is identical to that used in previous studies [20,21,22] and consists of 256 elements, each 7 mm in diameter. The elements are distributed randomly on a spherical surface shell with a radius of curvature of 130 mm and a diameter of 170 mm. The spacing between the elements vary from a minimum of 7.9 mm to a maximum of 9.4 mm. A hole of 38 mm in diameter in the center of the spherical shell is reserved to accommodate an ultrasound imaging transducer. The array has a low F-number of 0.76 and the range of the frequency of operation is 0.8 to 1.2 MHz. The layout of the array can be seen in Figure 1.

### 2.2. Parametric Studies

Simulations were performed to demonstrate the effect of ribs on the quality of HIFU focusing by calculating acoustic field distribution in both focal plane and plane of the ribs. The dependence of peak focal intensity on the ratio of beam width in the ribs plane and width of the ribs, the position of beam axis relative to the ribs, position of ribs relative to the transducer, width of the ribs, width of intercostal space, and number of ribs in the HIFU beam path was calculated. The spacing between ribs and the depth of sonication are important parameters impacting the focal quality and thus the patient safety and was studied in detail. High focal peak intensity and low level of secondary foci are desired and the parametric analysis in this work describes the effect of various array-rib configurations on the focus quality of HIFU beam.

### 2.3. Acoustic Field Modeling

Three-dimensional far-field pressure, P(x,y,z), of the phased array is calculated by summing the contribution from each circular element j which was calculated analytically at a point in the field with coordinates (x,y,z), which is at a distance r from the center of the element and at an angle θ from the line joining the center of the element with the geometric focus [23,24]:

(1) $$P\left(x,y,z\right)=\sum_{j}\frac{ip_{0}Z_{R}e^{i\left(kr+\varphi_{j}\right)}}{r}\frac{2J_{1}kasin\theta }{kasin\theta },$$

where $p_{0}$ is the pressure on elements surface and its value is equal to ρcv, where ρ is density of the medium, c is the speed of sound, and v is the vibration velocity at the surface of the transducer, a is radius of the transducer element, k is wave number, $Z_{R}=ka^{2}/2$ is the Rayleigh length, r is the distance from the element center to the field point, θ is the angle between the the lines joining the center of the element with the geometric focus and the field point, φ is the phase angle calculated using the pseudoinverse method discussed later, and $J_{1}$ is the first order Bessel function. The intensity distributions (I) in the plane of the ribs and in the focal plane were calculated using

(2) $$I\left(x,y,z\right)={\left|P\right|}^{2}/2\rho c,$$

where |P| is the pressure amplitude from all the elements.

The phases of the velocities at each element surface required to produce a single focus were calculated from the paths between the center of each element and the focus. For a particular focus configuration, the distribution of phases and velocity for each element is calculated using the pseudoinverse method [25]:

(3) $$u=H^{*t}{\left(HH^{*t}\right)}^{-1}p,$$

where $u=\left[u_{1},u_{2},\ldots \ldots ,u_{n}\right]$ is the set of complex surface velocity $u_{n}$ at each of the nth element, $p=\left[p_{1},p_{2},\ldots \ldots ,p_{m}\right]$ is the set of complex pressures $p_{m}$ at each of M target points and H, an m×n matrix with elements $h_{mn}=\frac{ip_{0}Z_{R}e^{i\left(kr_{mn}+\varphi_{n}\right)}}{r_{mn}}\frac{2J_{1}kasin\theta }{kasin\theta }$, is a forward propagation operator from the surface of the array to the set of control points and is the pressure due to element n at control point m, where m and n are number of control points and elements, respectively. $r_{mn}$ is the distance of the center of the $n_{th}$ element to the $m_{th}$ focus point and $\varphi_{n}$ is the excitation phase for each element. $H^{*t}$ is the complex conjugate of H and ${\left[\right]}^{t}$ denotes transpose. The amplitude and phase of each element were controlled independently, and the acoustic surface velocity and amplitude were modeled as uniform over the radiating surface of each array element.

### 2.4. Ribs Models

#### 2.4.1. 2D Parallel Idealized Ribs Model

**Figure 2:**
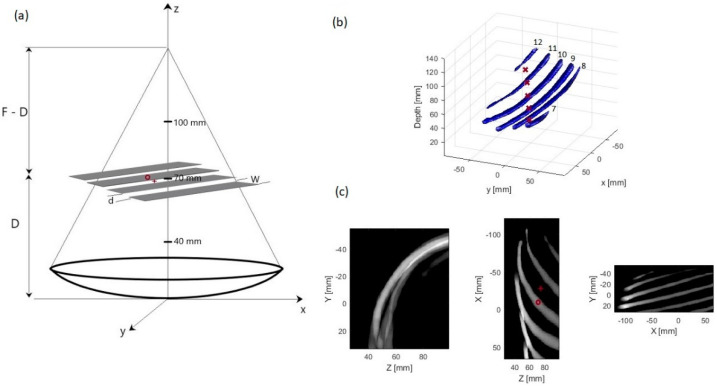
(**a**) Sketch of the geometric representation of the idealized rib cage and the transducer for the case when the ribs are positioned at a depth of 70 mm from the array center and the array axis passes through the center of one of the ribs, (**b**) 3D model of the patient-specific rib cage showing ribs 7–12. Five different depths of the 3D model used in the simulation are marked (×), (**c**) 2D slices of the rib cage model. (D: depth of the ribs plane from the array center, d: edge–edge spacing of the ribs, W: width of a rib, F: focal length of the array, o: center of a rib, +: center of intercostal spacing).

Three idealized rib geometries were used to study the effect of ribs with varied dimensions. These geometries consisted of parallel strips of different width and spacing between them. The strips were 10 mm, 14 mm and 20 mm wide, and the spacing between them was 20 mm, 16 mm, and 10 mm, respectively. Moreover, these strips were positioned in such a way that the focal point (F−D) was 90 mm, 60 mm, and 30 mm behind the ribs, i.e., the strips were placed at depths of 40 mm, 70 mm, and 100 mm from the array center. For each of these configurations, two scenarios were investigated: when the array axis passes through the middle of one of the ribs and when it passes through the center of an intercostal space. A sketch of the geometry of array-ribs configuration is shown in Figure 2.

#### 2.4.2. 3D Patient-Specific Ribs Model

3D anatomical tissue model was generated using anonymized human CT images, by importing the CT image set into a 3D Slicer using embodi3D, open source software platform, for segmentation of the bone. Ribs 7 to 12 of the right side of the model were severed from the spine due to its close proximity to the liver, and imported as STL file into MATLAB 2018a (The MathWorks, Inc., Natick, MA, USA) for modeling where it was meshed and then voxelized for use in ray tracing and acoustic modeling algorithms. The rib cage was positioned at five different depths from the center of the transducer ranging from 40 mm to 100 mm and was aligned such that the beam axis either passed through one of the ribs or the middle of intercostal spacing.

### 2.5. Ray Tracing

**Figure 3:**
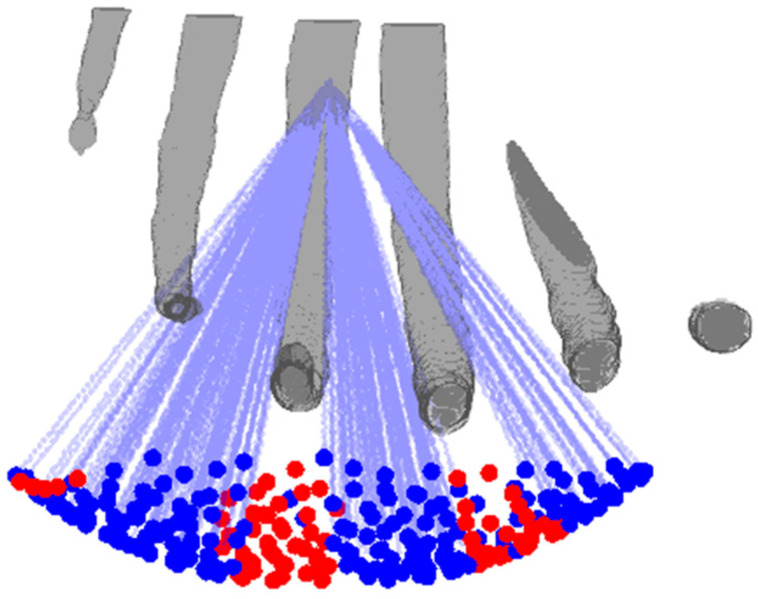
Configuration of the anatomical ribs with respect to the array. Rays are traced from the array elements to the focus, and the elements blocked by the ribs are deactivated and shown in red.

The medium of interest was divided into three-dimensional voxels and the ray tracing approach was used to determine the elements that are being shadowed by the ribs. A ray from the center of each element was traced to the intended focus. If the ray passes through any of the ribs, the element is deactivated by setting its amplitude to zero otherwise the element is set to active. Figure 3 shows the array-rib configuration and the rays traced from the active elements to the geometric focus of the array.

## 3. Results

### 3.1. Parallel Idealized 2D Ribs

**Figure 4:**
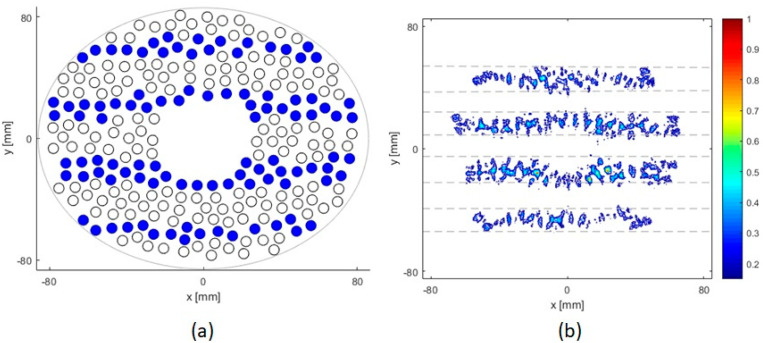
(**a**) Distribution of active elements of the array. Filled circles denote active elements. The number of active elements is 100. (**b**) Predicted intensity distribution in the ribs plane for a single focus generated at the center of curvature of the array (0, 0, 130) mm when the rib phantom was placed at 40 mm depth from the center of the array. Striped intensity distribution suggests that the energy is delivered only through the spaces between the ribs.

The intensity distributions in the focal plane and plane of the ribs were calculated for a single focus in the presence of parallel idealized 2D ribs. Figure 4 shows the intensity distribution in the plane of the ribs in the xy plane at a depth of 40 mm from the array center. It is seen that the intensity distribution is striped in accordance to the parallel strips and most of the energy is delivered intercostally. To calculate intensity in the focal plane, the acoustic power of the phased array was set arbitrarily to 15 W which is equivalent to an input surface intensity of 0.15 W/cm^2^. When all the elements are active with uniform amplitude distribution and there are no ribs in place, the peak intensity in the focal plane in water is 387.6 W/cm^2^. Peak intensity in the focal plane in the presence of ribs is reduced between 22.7% and 70.9% depending on the position and orientation of the rib cage as shown in Table 1. The focal intensity reduces with the increasing width of the ribs as more elements are deactivated. The lowest peak focal intensity is observed when the width of the ribs is twice the spacing between them, thus shading more elements.

**Figure 5:**
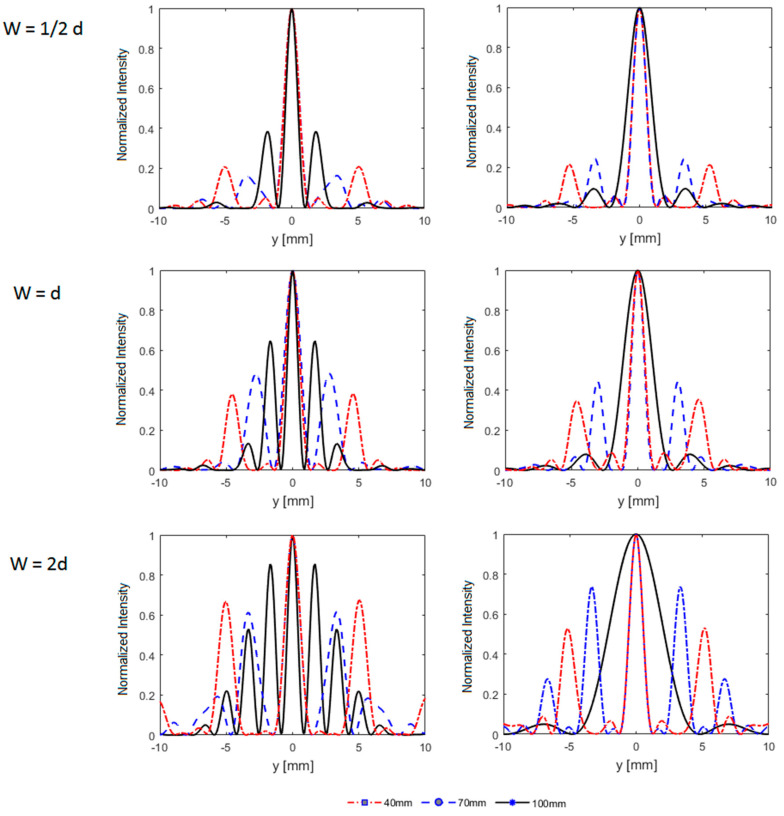
Intensity distribution in the focal plane for different rib phantoms (W = 1/2d, W = d, W = 2d) where W is the width of the ribs and d is the edge–edge spacing between them, when the phantoms are positioned at a depth of 40 mm (red dotted line), 70 mm (blue dashed line), and 100 mm (black solid line) from the center of the array, and when the beam axis passes through one of the ribs (**left column**) and intercostal space (**right column**).

Focus splitting into grating lobes is another important phenomenon that takes place in the presence of the rib cage. Figure 5 shows that the focus is split into three or more peaks depending on the position and geometry of the rib phantom. It is observed that focus quality depends on the ratio of the width of the ribs to the intercostal space, the position of the beam axis relative to the intercostal space, and the distance of the rib phantom from the array transducer. Table 1 summarizes the parameters of intensity distribution and the focus splitting for the parallel idealistic rib configurations mentioned before. As can be seen in Figure 5 and Table 1, the closer the rib phantom is to the transducer, the higher will be the number of ribs in the beam aperture creating more grating lobes which are farther from the main lobe. The side lobes get closer to the main lobe when the spacing between the ribs and the transducer is increased. When the rib phantom is close to the focus (z = 100 mm), large grating lobes are observed if the main beam hits a rib and low grating lobes if the main beam is aligned with an intercostal space, although the size of the focus increases notably. Significantly high side lobes are observed for the ribs with width twice the spacing between them. For ribs with width half the spacing, the side lobes are lowest with maximum peak focal intensity. The number and level of side lobes increase with increasing the width of the ribs while the peak focal intensity decreases.

### 3.2. Anatomically Correct 3D Ribs Model

**Figure 6:**
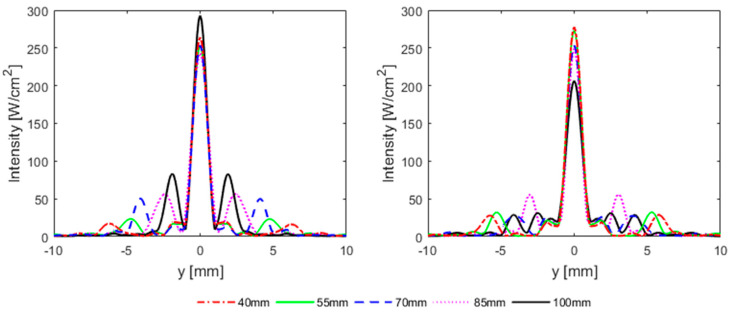
Horizontal intensity distribution in y-direction when the ribs are at 40 mm (red dotted line), 55 mm (green solid line), 70 mm (blue dashed line), 85 mm (magenta dotted line), and 100 mm (black solid line) from the array center and the beam axis passes through the middle of rib 9 (**left**), middle of intercostal spacing between ribs 9 and 10 (**right**).

In the previous section and the previous literature [7,8], the focal quality was evaluated for idealistic 2D ribs, which may not be representative of the situation with anatomically realistic curved ribs. Simulations were performed for the more realistic 3D rib geometry for cases where the rib cage from the array center was 40 mm, 55mm, 70 mm, 85 mm, and 100 mm from the array center, and the beam axis either passed through the middle of rib 9 or the intercostal spacing between ribs 9 and 10. The results are shown in Table 2 and Figure 6.

**Figure 7:**
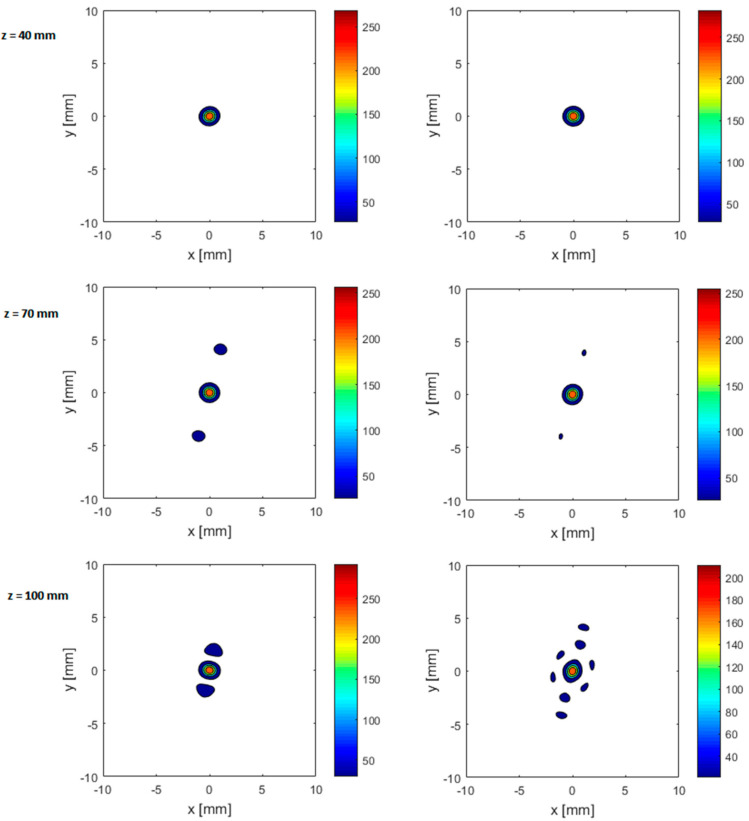
Intensity distribution and focus splitting in the transverse focal plane, for anatomically correct ribs positioned at a distance of 40mm (**top row**), 70 mm (**middle row**), and 100 mm (**bottom row**) from the array center and the beam axis passes middle of rib 9 (**right column**) and middle of intercostal space between ribs 9 and 10 (**right column**). Color bar indicates intensity in [W/cm^2^].

As shown in Figure 6, irrespective of the alignment of the beam axis, a single sharp focus is generated at the target position when the rib cage is near the transducer at a distance of 40 mm from the array center, whereas secondary foci appear when the rib cage is moved away from the transducer. At 70 mm depth, the secondary foci have high amplitude when the beam axis passes through the middle of the rib and with half the amplitude when the beam axis passes through intercostal spacing. At a depth of 100 mm, the secondary foci are as close as 1.9 mm on either side of the main beam when the beam axis aligns with the rib, and the main focus would split into many foci surrounding it in a circle with a diameter of 5 mm when the beam is aligned with the intercostal spacing (Figure 6 and Figure 7). It can also be noted that the side lobes for 3D realistic ribs are much lower than the idealistic 2D ribs (as can be seen from comparing Figure 5 and Figure 6). It should further be noted that secondary foci with an amplitude of less than 0.1 I_peak_ are generally considered safe. Figure 7 shows intensity distribution and position of side lobes in a transverse plane across the geometric focus of the transducer.

#### 3.2.1. Effect of Axial Steering on Focus Quality

The transcostal performance of the phased array was further analyzed by steering the focus in the therapeutic field from a depth of 110 mm to 150 mm along the array axis to assess the best geometric configuration of the rib cage relative to the phased array in terms of peak intensity and energy in the secondary foci. As before, the rib cage was positioned at five different heights from the center of the phased array ranging from 40 mm to 100 mm and was aligned such that the array axis either passed through the center of the rib or middle of the intercostal spacing. The focus point was steered from 110 mm to 150 mm with a step of 10 mm. No transverse steering was performed.

**Figure 8:**
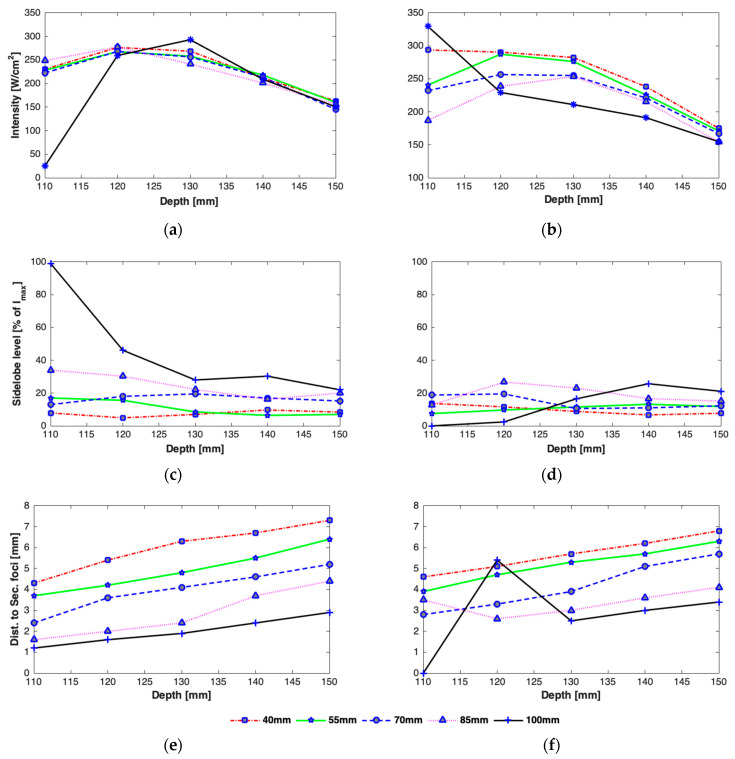
Effect of axial steering on the peak focal intensity (**a**,**b**), magnitude of secondary foci (**c**,**d**), and spacing between the main focus and secondary foci (**e**,**f**), when the beam axis is aligned with one of the ribs (**a**,**c**,**e**) and center of intercostal spacing (**b**,**d**,**f**), for rib cage positioned at 40 mm, 55 mm, 70 mm, 85 mm, and 100mm and for a nominal input acoustic power of 15 W.

The effect of changing the focal depth as well as distance of the rib cage from the transducer is shown in Figure 8. It is observed that intensity tends to be at a maximum at and near the geometric focus irrespective of the position of the rib cage (Figure 8a,b). A reduction in the peak focal intensity is observed when the array is focused beyond the geometric focus. A high dependency of the peak focal intensity on the transverse position of the rib cage is observed when the rib cage is positioned closer to the focus, i.e., z = 100 mm. If the array-rib configuration is such that the beam axis passes through the middle of a rib and the focus point is only 10 mm behind the ribs, a very low focal intensity (24.7 W/cm^2^) is achieved due to the shadowing of the focus point by the ribs (Figure 8a), whereas maximum focal intensity (329.5 W/cm^2^) is observed for the same focal depth when the array axis passes through the middle of the intercostal space (Figure 8b). However, the peak intensity drops significantly when the focus is steered away from the ribs when the ribs are located at a depth of 100 mm and when the beam axis passes through an intercostal space.

The effect of array-rib configurations on the side lobe level was also studied in detail for different axial depths. As can be seen in Figure 8, secondary foci with higher amplitudes appear when the focus point is very close to the rib cage and the beam axis aligns with a rib (Figure 8c). In the worst case, the amplitude of the peak intensity in the secondary lobe equals that in the main beam when the array is focused at a focal depth of 110 mm, the rib cage is positioned at a depth of 100 mm from the array center, and the beam axis passes through the rib. Conversely, when the array axis passes through the middle of an intercostal spacing and the rib cage is closer to the focus point, secondary foci with a small amplitude are formed (Figure 8d). The amplitude of secondary foci is thus dependent not only on the depth of the rib cage from the array but also the alignment of the array with respect to the rib cage. The intensity in secondary foci generally decreases by increasing the distance between the focus point and the rib cage. There is also an effect on the spacing between the main beam and secondary foci, which varies linearly with the depth of the rib cage (Figure 8e,f). The larger the distance between the focal point and the rib cage, the farther apart will be the main and secondary foci.

#### 3.2.2. Effect of Transverse Steering on Focus Quality

In addition to axial steering, the focal quality was also assessed when steering the focus point in both transverse directions relative to the rib orientation. The focus was steered 15 mm each side of the geometric center of the array in the focal plane with a step size of 5 mm. The array-rib configurations were kept same, i.e., the rib cage was positioned at five different heights ranging from 40 mm to 100 mm and the array axis passed either through the middle of the rib or the intercostal space. No axial steering was performed.

**Figure 9:**
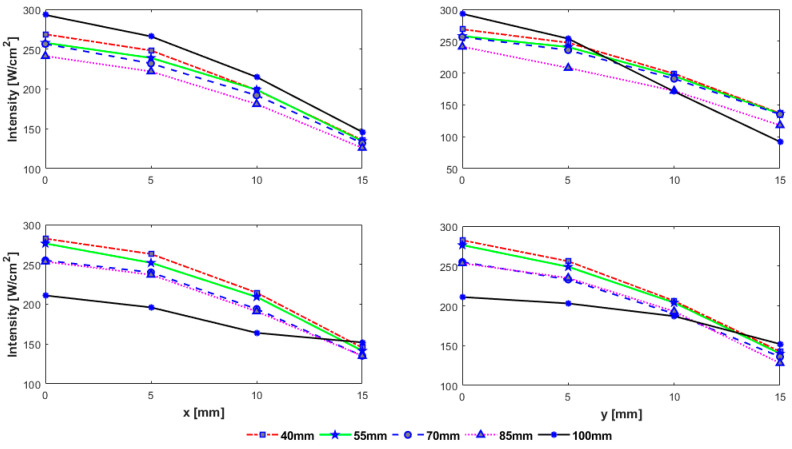
Change in the peak focal intensity with transverse steering for rib cage positioned at 40 mm, 55 mm, 70 mm, 85 mm, 100 mm at a nominal acoustic power of 15 W and when the array axis crosses center of the rib (**top row**), center of intercostal space (**bottom row**); steered in the x-direction (**left**), y-direction (**right**).

**Figure 10:**
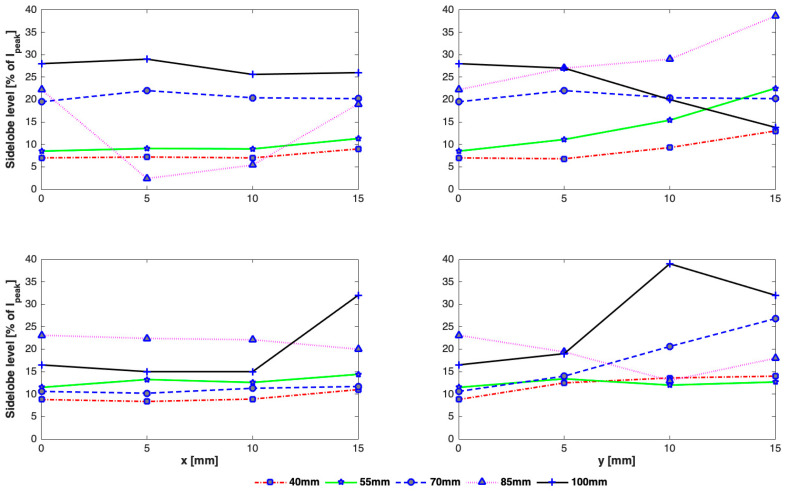
Change in the magnitude of secondary foci with transverse steering for rib cage positioned at 40 mm, 55 mm, 70 mm, 85 mm, 100 mm and when the array axis crosses center of the rib (**top row**), intercostal space (**bottom row**), steered in x-direction (**left column**), y-direction (**right column**).

**Figure 11:**
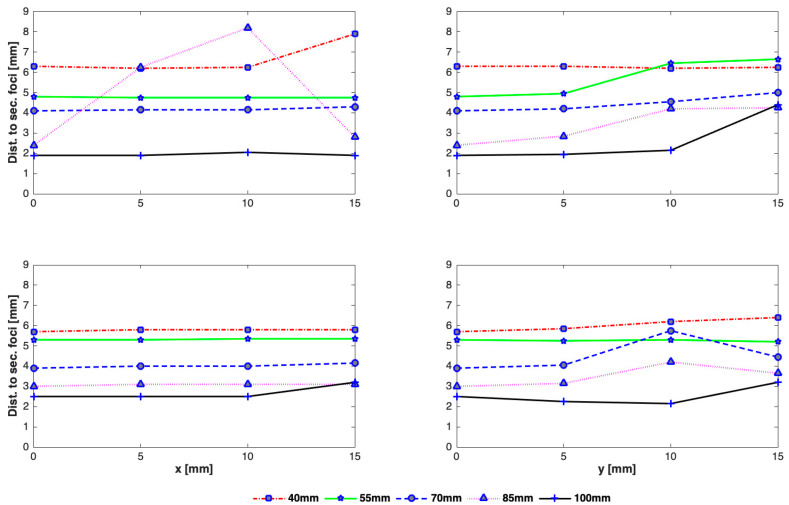
Effect on the distance to secondary foci from main focus with transverse steering for rib cage positioned at 40 mm, 55 mm, 70 mm, 85 mm, 100 mm and when the array axis crosses center of the rib (**top row**), intercostal space (**bottom row**), steered in x-direction (**left column**), y-direction (**right column**).

As expected, the intensity is highest along the axis and drops down away from it irrespective of the position of the rib cage and the alignment of the beam axis. There is variation in the peak intensity for different heights of the rib cage. A significant disparity in the peak focal intensity is observed for the focus point near the rib cage. For example, when the rib cage is positioned at a depth of 100 mm, the intensity at focus point would be highest if the array axis passes through a rib and lowest if it passes through intercostal space as can be seen in Figure 9. From Figure 10, it can be seen that steering in either lateral or elevation directions does not significantly affect secondary lobe level, however, intensity in secondary foci was low when the rib cage was positioned at a depth of 40 mm and high when positioned at 100 mm. The intensity in secondary foci thus increases with the distance between the transducer and the rib cage. The spacing between the main focus and the secondary foci does not vary while steering up to 10 mm in any transverse direction (Figure 11). There is a significant variation in the spacing when the rib cage is positioned at different heights. Secondary foci are closest to the main focus when the rib cage is at 100 mm depth and move farther away when the transducer gets closer to the rib cage (Figure 8). The parameters associated with intensity distribution and focus splitting for targeting at the geometric focus of the array are summarized in Table 2.

**Figure 12:**
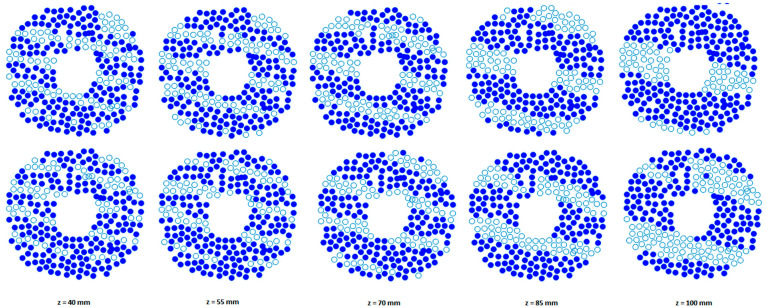
Active elements when the ribs are positioned at different heights [40,55,70,85,100] mm. Blue filled circles indicate elements that are turned ON for HIFU and unfilled circles indicate elements which are blocked by ribs and turned OFF. When the array axis crosses: middle of rib 9 (**top row**), middle of the space between ribs 9 and 10 (**bottom row**).

Figure 12 shows the distribution of active elements that are turned on for targeting the beam at the geometric focus and when the array axis passes through the rib and through the intercostal space for different positions of the rib cage. It is seen that different sets of elements are active for different array-rib configurations.

## 4. Discussion

The use of a random phased array to produce large volumes of ablated tissue by scanning a single focus or set of multiple foci has been described by several authors [7,24,26,27,28,29]. Preliminary investigations to customize the acoustic field of a 256-elements random phased array to enable ablation of liver was previously demonstrated by our group [22]. We have also demonstrated the dual nature of imaging and therapy of the spherical random phased array, thus providing a platform to image the strong aberrating structures such as rib cage in real time and tailor the acoustic field to achieve high peak focal intensity while avoiding heating of the ribs [21]. Ribs in the HIFU beam path not only get heated, due to their high absorption capabilities, leading to skin burns, but can also distort the focus quality significantly by generating secondary hotspots which may be harmful to the adjacent healthy tissue and critical structures. The estimate of decrease in the HIFU intensity in the focal plane and increase of intensity in secondary maxima due to the presence of ribs is thus very important for practical applications. This study employed quantitative acoustic simulations to estimate the effect of idealized as well as realistic ribs geometries on the delivery of ultrasound from a random phased array through the ribs. Focus quality and delivery of ultrasound energy through the ribs was studied for different array-rib configurations and different depths of the ribs.

While the intensity in the main focus is reduced due to the need to turn-off the elements that hit the ribs and grating lobes are generally formed due to its periodic structure, intensity distribution and focus deterioration highly depend on the orientation of the rib cage, its distance from the transducer, depth of sonication behind the ribs, and width of the ribs and the spacing between them. Wider ribs shadow more elements causing not only reduction in the focal intensity but also cause the creation of multiple significantly high side lobes. The reduction in the peak intensity was in the range of 22–29%, 44–50%, and 53–71%, whereas the increase in the magnitude of side lobes was in the range of 18–97%, 8–64%, and 10–38% in the presence of 2D idealistic ribs with widths twice the intercostal space, same as intercostal space, and half the intercostal space, respectively. For a realistic 3D rib cage, the reduction in the peak intensity varied from 54% to 75% and the magnitude of secondary lobes increased in the range of 7–28% for various depths of the rib cage from the transducer. A detailed analysis is shown in Figure 7, Figure 8, Figure 9, Figure 10 and Figure 11. In practical applications, the focus is steered both in axial and lateral directions and the distance between the transducer and ribs is adjusted according to the depth of sonication in the liver. In the previous studies [7,11,16], this effect was not taken into consideration and only a fixed distance between transducer and rib cage was considered. Usually, the target is set initially at the geometric focus of the array due to the high gain of the array at the focus; however, both axial and transverse steering are necessary to scan and ablate the entire tumor volume. The distance between the array and the ribs thus plays an important role in the intensity level of both the main focus and the secondary foci. Secondary maxima with higher intensity are noticed when the distance between the transducer and rib cage is increased (Figure 8). Thus, secondary lobes can be significantly suppressed if the distance between the array and ribs is reduced. It is noticed that secondary lobes can be kept at below 20% of the peak focal intensity when the ribs are positioned at a depth of 70 mm or less (i.e., a maximum depth of 3 cm from the surface of the array) and when the beam axis crosses one of the ribs. If the rib cage is at a position deeper than 70 mm, then lower side lobes are observed when the beam axis crosses the intercostal space. Thus, for heating a shallow tumor closer to the rib cage, the beam axis should be aligned with the intercostal spacing in order to suppress side lobes. However, the secondary foci will be farther from the main beam when the array is closer to the ribs and vice versa. The separation between the main beam and secondary foci increases with increasing the spacing between the transducer and the rib cage and by steering the beam away from the transducer (Figure 8). While the focal intensity gain reduced with transverse steering in the range of 23–68% for the ribs positioned at different depths and for steering the beam up to 15 mm either side of the axis (Figure 9), the effect on the side lobe level and their distance from the main beam did not vary significantly (Figure 10 and Figure 11). By careful observation of the results of both the idealistic and realistic ribs, it is observed that the effect of beam alignment is more pronounced when the target is closer to the rib cage. Thus, if the HIFU beam is targeted in the liver at a depth of 6 cm or less behind the rib cage, high intensity in the main focus and low intensity in the secondary lobes will be achieved only when the beam axis is aligned with the intercostal space.

In this work, simulations are performed in a homogeneous medium. While the acoustic properties of different soft tissues do not alter significantly, tissue heterogeneity may have a further effect on the focus quality. Nevertheless, the present study provides a detailed quantitative and qualitative insight into the acoustic field of transcostal HIFU. In future, full wave three-dimensional modeling of ultrasound propagation in heterogeneous tissue will be performed considering both the diffraction effect of the ribs and the effect of nonlinear behavior on the focus quality. Acoustic field distribution on the surface of the ribs as well as at the focus would also be experimentally validated with the human rib cage in water and hydrophone measurements using the available random phased array. By utilizing the imaging capabilities of the random phased array, ribs will be detected and imaged in real time and the correction methods discussed here will be employed to avoid heating of the ribs while delivering enough energy to the target tumor.

The simulations performed here calculate the acoustic intensity, whereas the tissue effect is dependent on the consequent temperature rise whose distribution may differ due to blood cooling and thermal conduction. To fully evaluate this, the simulation will need to be extended to incorporate the bio-heat equation. However, useful observations can be made of the effect of the ribs on tissue heating from the present study. For applications of ultrasound with short duration, the temperature profiles will be similar to the intensity profiles shown here. Moreover, the effect of tissue cooling will tend to smooth out the focal peaks so the close grating lobes may merge with the main focus resulting in an enlarged ablated tissue volume. The effect of rib sparing on the delivered focus may not significantly alter the desired therapy but will protect the ribs.

## 5. Conclusions

The effect of a realistic human rib cage model on the HIFU beam was investigated theoretically. Determining this effect is important as it influences not only the efficacy of the HIFU system to ablate tumor but also the safety of this method to perform transcostal ablation. Acoustic simulations were performed to comprehensively understand the ultrasound beam patterns affected by the rib structure and the changes caused in the peak focal pressure. The dimensions of the ribs (width and spacing between the ribs), position (various depths), and alignment (beam axis through ribs and intercostal space) of the transducer relative to the ribs were used to calculate the effect on focus quality by determining the peak intensity at the focus, intensity in the side lobes, number and positions of side lobes, and intensity in the ribs plane. Parametric modeling studies demonstrated that the ribs can be avoided from heating while preserving the focus quality when the position and orientation of the rib cage is known. The results presented here may contribute to the treatment planning strategy to ensure efficient and safe therapy of the liver tumor with high intensity focused ultrasound.

## Figures and Tables

**Table 1:** Intensity distribution and focal quality for different ribs geometry configuration positioned at three different depths (40 mm, 70 mm, 100 mm) from the array center, when the beam axis passes through the middle of one of the ribs (case I) and middle of the intercostal space (case II). Strip width is the width of the strips in terms of the intercostal edge–edge spacing d. The results shown are for a single focus targeted at the geometric focus of the array.

Case	Depth [mm]	Ribs Width [mm]	I_peak_ [W/cm^2^]	Number of Foci	Distance to Secondary Foci [mm]	Secondary Foci Level [% of I_peak_]	Number of Active Elements
I	40	½ d	226.20	3	5	20	149
		d	195.83	3	4.5	38	129
		2d	92.6	5	5 and 10	67 and 18	61
	70	½ d	247.45	3	3.5	20	149
		d	179.13	3	2.7	38	129
		2d	113.86	7	3.4 and 5.7 and 9	67 and 18	61
	100	½ d	274.78	3	1.8	38.5	181
		d	192.80	5	1.7 and 3.3	64 and 13.2	127
		2d	113.86	7	1.7 and 3.3 and 5	97.3 and 60 and 25	75
II	40	½ d	236.82	3	5.3	20.5	156
		d	176.10	3	4.6	33.7	116
		2d	110.82	3	5.1	50	74
	70	½ d	236.82	3	3.5	10	137
		d	186.73	3	3.1	43	123
		2d	112.34	5	3.4 and 6.8	70.5 and 22.2	74
	100	½ d	208	3	3.5	10	137
		d	171.54	3	4	8	113
		2d	88.05	1	0	0	58

**Table 2:** Intensity distribution and focal quality for anatomical ribs, when the array axis passes through the ribs (case I) and the intercostal space (case II). The results shown are for a single focus targeted at the geometric focus of the array.

Case	Depth [mm]	I_peak_ [W/cm^2^]	Number of Foci	Distance to Secondary Foci [mm]	Secondary Foci Level [% of I_peak_]	Number of Active Elements
I	40	268.70	3	6.3	7	177
	55	258	3	4.8	8.5	167
	70	256.56	3	4.1	19.45	169
	85	241.3	3	2.4	22.2	160
	100	293	3	1.9	28.18	193
II	40	282.37	3	5.7	8.8	186
	55	276.3	3	5.3	11.5	180
	70	255.04	3	4	10.6	168
	85	253.5	5	3	23.1	164
	100	211.02	5	2.5 and 4.1	16 and 3.5	139
